# Integration of zebrafish fin regeneration genes with expression data of human tumors *in silico* uncovers potential novel melanoma markers

**DOI:** 10.18632/oncotarget.12257

**Published:** 2016-09-26

**Authors:** Martin Hagedorn, Géraldine Siegfried, Katarzyna B. Hooks, Abdel-Majid Khatib

**Affiliations:** ^1^ University of Bordeaux, Talence F-33405, France; ^2^ INSERM, LAMC, U1029, Talence F-33405, France; ^3^ INSERM, BMGIC, U1035, Bordeaux F-33000, France

**Keywords:** regeneration, melanoma, angiogenesis, BAMBI, FK506-binding proteins

## Abstract

Tissue regeneration requires expression of a large, unknown number of genes to initiate and maintain cellular processes such as proliferation, extracellular matrix synthesis, differentiation and migration. A unique model to simulate this process in a controlled manner is the re-growth of the caudal fin of zebrafish after amputation. Within this tissue stem cells differentiate into fibroblasts, epithelial and endothelial cells as well as melanocytes. Many genes implicated in the regeneration process are deregulated in cancer. We therefore undertook a systematic gene expression study to identify genes upregulated during the re-growth of caudal fin tissue. By applying a high stringency cut-off value of 4-fold change, we identified 54 annotated genes significantly overexpressed in regenerating blastema. Further bioinformatics data mining studies showed that 22 out of the 54 regeneration genes where overexpressed in melanoma compared to normal skin or other cancers. Whereas the role of TNC (tenascin C) and FN1 (fibronectin 1) in melanoma development is well documented, implication of MARCKS, RCN3, BAMBI, PEA3/ETV4 and the FK506 family members FKBP7, FKBP10 and FKBP11 in melanoma progression is unclear. Corresponding proteins were detected in melanoma tissue but not in normal skin. High expression of FKBP7, DPYSL5 and MDK was significantly associated with poor survival. We discuss a potential role of these novel melanoma genes, which have promising potential as new therapeutic targets or diagnostic markers.

## INTRODUCTION

Identification of reliable and specific cancer biomarkers, which would help to diagnose, classify and predict disease outcome is still a challenging task. This is particularly true for malignant melanoma (MM), because only a small quantity of tissue is available at the time of diagnosis. Routinely, melanoma are classified using histological parameters such as tumor thickness and ulceration, serum levels of lactate dehydrogenase (LDH) or staining intensity for S100B protein [1].

During melanoma growth and progression in the adult, developmental genes important for melanocyte differentiation are re-activated. Identification of more of these genes in an adequate *in vivo* experiment might uncover novel melanoma markers. During zebrafish fin regeneration after amputation, melanocyte precursor cells give rise to pigment-producing cells in the new blastema tissue, thereby mimicking the process of melanocyte differentiation in a short time frame [2]. Using microrarrays we compared normal fin tissue to regenerating blastema after amputation and investigated upregulated genes in the Oncomine cancer database [3] to identify those increased in melanoma. Interestingly, we found that 40% of the blastema-upregulated genes are also overexpressed in melanoma. These include known melanoma-promoting proteins such as fibronectin and tenascin C [4, 5], but also novel promising candidates such as BAMBI, a negative regulator of TGF-beta signaling [6]. The role of MARCKS in cell motility during melanoma progression is poorly understood [7]. FK506-binding proteins bind to immunosuppressive drugs such as Tacrolimus (FK506), which is used to treat patients after organ transplantation. Members of this family have been shown to be promising new targets in anticancer therapy [8]. FK506 family member FKBP51 promotes melanoma metastasis *in vivo* [9] but nothing is known about the role of FKBP7, 10 and 11 in melanoma growth. Data provided in this paper also show that high expression of FKBP7 is associated with poor survival.

We further studied overexpression at the protein level of these candidates in melanoma lesions. No or very low expression was observed in control skin. Additional co-expression studies with defined gene signatures using Oncomine suggest an association of BAMBI and FKBP10 with the angiogenic process in melanoma and with regeneration after HRAS induced cell transformation, respectively. Taken together, our results suggest that some of the genes induced in physiological regeneration hold the potential to play roles in melanoma malignancy and warrant further functional *in vitro* and *in vivo* studies.

## RESULTS

### Microarray and oncomine expression data

**Figure 1:**
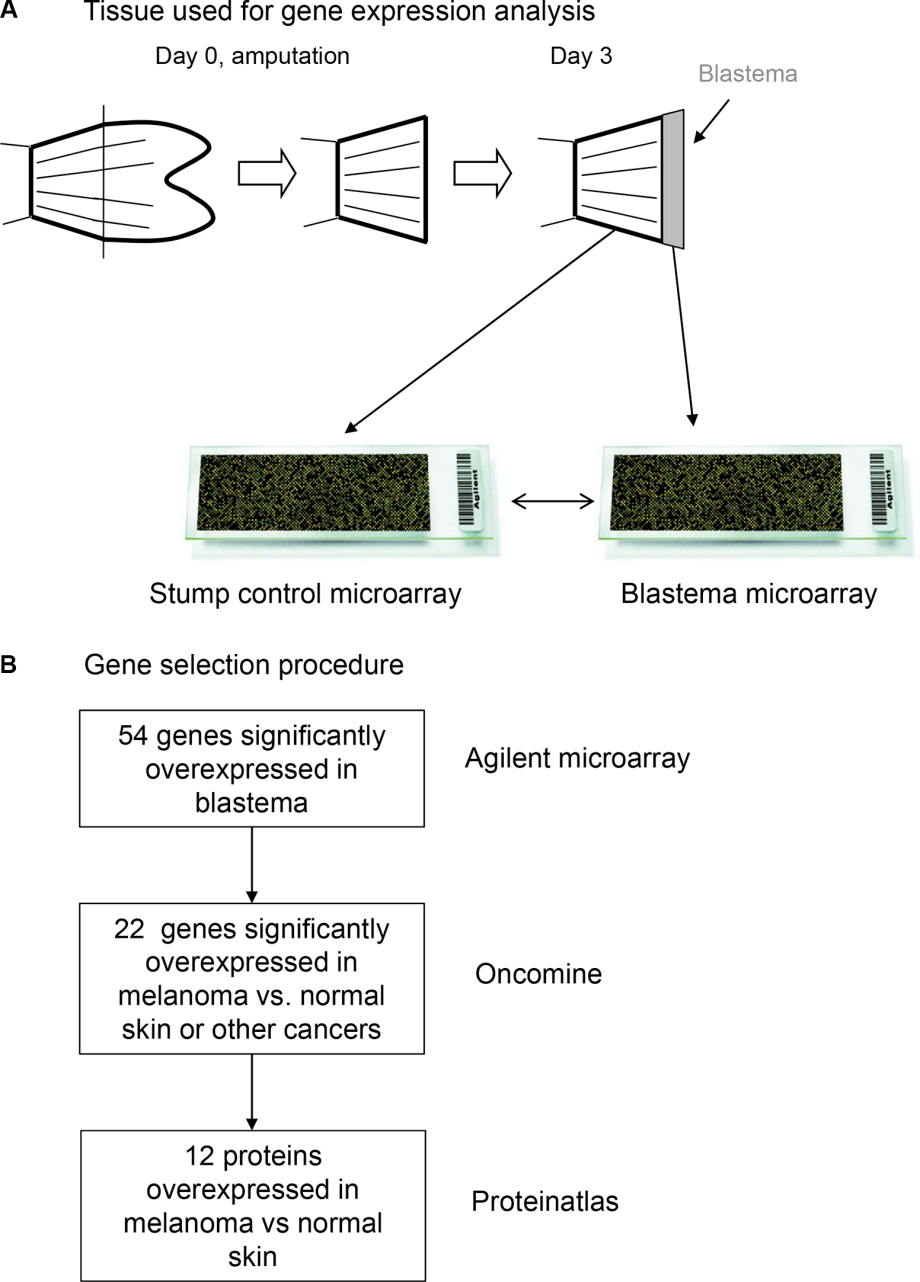
(**A**) Regenerating zebrafish tissue used for gene expression analysis. (**B**) Gene selection pipeline leading to the identification of novel melanoma overexpressed genes.

**Figure 2:**
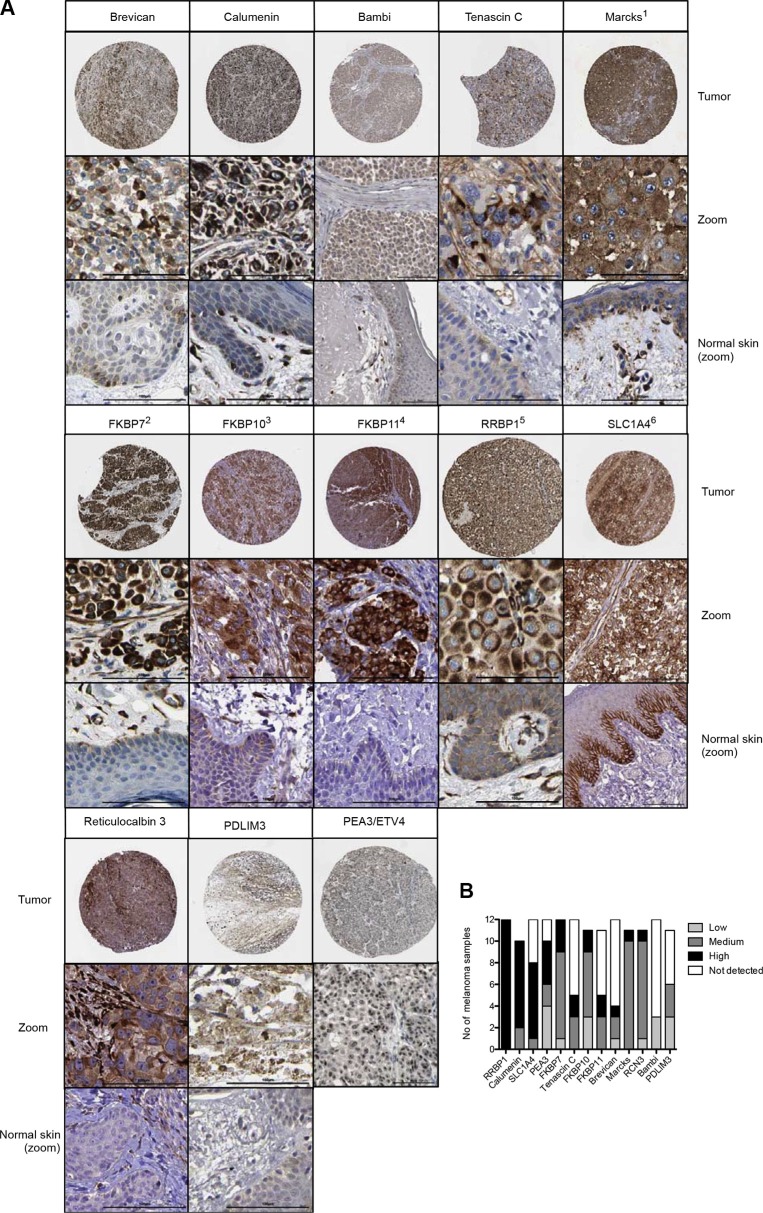
(**A**) Gene products for melanoma overexpressed genes were screened for staining in patient melanoma using the Protein Atlas database. The circle for each protein shows the overview of the stained tumor tissue, the square directly below a magnification of a representative region of the tumor (zoom, bar = 100 μm), and the square below a magnified section of normal skin (bars = 100 μm). 1: myristoylated alanine-rich protein kinase C substrate, 2: FK506 binding protein 7, 3: FK506 binding protein 10, 65 kDa, 4: FK506 binding protein 11, 19 kDa, 5: ribosome binding protein 1, 6: solute carrier family 1 (glutamate/neutral amino acid transporter), member 4. (**B**) Summary of staining intensities for indicated proteins were inferred from Protein Atlas data.

To find genes strongly induced in regenerating blastema we applied a stringent 4-fold-change cut off to significantly regulated genes in our microarray experiments (Figure 1). 54 genes detected by 71 probes fulfilled this criterion (Table 1). Since melanocyte differentiation takes place during blastema development and genes participating in this process are deregulated in MM initiation and progression (reviewed in [2]), we determined expression levels of our regeneration genes in public available datasets of melanoma transcriptome studies using Oncomine. 40 percent of the regeneration genes were overexpressed in melanoma, either compared to normal skin (12/54, Figure 2A) or compared to other cancers (10/54; Table 1).

**Table 1:** Fifty-four genes (detected by 71 probes) were significantly overexpressed at least 4-fold in regenerating blastema compared to normal fins

Gene Symbol	Gene Name	Up in blastema (foldchange)	*P*-value	*q*-value (adj *p*-value)	Melanoma vs normal / other cancers (foldchange)	*P*-value
adam8a	a disintegrin and metalloproteinase domain 8a (adam8a)	35.53	1.55E-10	2.46E-06		
bambi	BMP and activin membrane-bound inhibitor (Xenopus laevis) homolog	4.69	5.54E-06	4.09E-04	5.556	4.21E-05
**bambi**	**BMP and activin membrane-bound inhibitor (Xenopus laevis) homolog**	**4.69**	**5.54E-06**	**4.09E-04**	**10.761**	**8.14E-25**
BC049329	mRNA similar to exportin 1, CRM1 homolog (yeast)	8.89	1.55E-09	4.05E-06		
bcan	brevican (bcan)	5.36	5.34E-08	2.13E-05	22.309	7.56E-19
bcl2l10	BCL2-like 10 (apoptosis facilitator) (bcl2l10)	5.87	1.24E-08	9.48E-06		
bcl2l10	BCL2-like 10 (apoptosis facilitator) (bcl2l10)	8.52	3.22E-09	5.62E-06		
calua	calumenin a (calua)	10.29	9.67E-10	3.90E-06	24.953	4.70E-7
col10a1	collagen, type X, alpha 1 (col10a1)	24.23	1.18E-05	6.39E-04	5.614	4.48E-5
col10a1	collagen, type X, alpha 1 (col10a1)	28.45	6.77E-06	4.62E-04	5.614	4.48E-5
col1a1	collagen, type I, alpha 1 (col1a1)	9.47	3.74E-05	1.30E-03		
col1a1	collagen, type I, alpha 1 (col1a1)	20.28	1.21E-06	1.63E-04		
crip2	cysteine-rich protein 2 (crip2)	11.54	1.62E-07	4.05E-05		
**cygb1**	**cytoglobin 1 (cygb1)**	**27.61**	**7.15E-09**	**7.26E-06**	**5.76**	**2.76E-10**
**cygb1**	**cytoglobin 1 (cygb1)**	**36.88**	**2.21E-09**	**4.60E-06**	**5.76**	**2.76E-10**
dachc	dachshund c (dachc)	6.39	1.11E-08	8.95E-06		
dkk1	dickkopf 1 (dkk1)	4.08	3.03E-06	2.86E-04		
dlx4a	distal-less homeobox gene 4a (dlx4a)	4.56	1.10E-07	3.23E-05		
dlx4a	distal-less homeobox gene 4a (dlx4a)	4.77	1.08E-07	3.21E-05		
dpysl5a	dihydropyrimidinase-like 5a (dpysl5a)	7.65	7.17E-08	2.49E 05		
egfl6	EGF-like-domain, multiple 6 (egfl6)	10.45	1.48E-09	4.05E-06		
eno2	enolase 2 (eno2)	7.00	1.61E-07	4.05E-05	7.17	1.43E-5
ENSDART00000046209	Acbd7 protein	7.09	3.48E-06	3.12E-04		
**fabp7a**	**fatty acid binding protein 7, brain, a**	**19.77**	**1.05E-09**	**3.90E-06**	**7.192**	**3.92E-8**
fkbp10	Fkbp10 protein (Fragment)	11.36	2.20E-09	4.60E-06	15.183	8.77E-6
fkbp11	FK506 binding protein 11 (fkbp11)	15.02	1.10E-08	8.95E-06	2.424	5.93E-6
fkbp11	FK506 binding protein 11 (fkbp11)	15.05	3.29E-08	1.71E-05	2.42	0.00000593
fkbp7	FK506 binding protein 7 (fkbp7)	8.90	1.47E 07	3.82E 05	3.214	5.47E-18
fn1b	fibronectin 1b (fn1b)	5.43	2.52E-06	2.58E-04	5.654	6.59E-9
fn1b	fibronectin 1b (fn1b)	9.14	7.69E-07	1.20E-04	5.65	6.59E-09
**fstl1**	**follistatin-like 1 (fstl1)**	**5.33**	**9.83E-06**	**5.74E-04**	**3.823**	**7.45E-10**
hrh3	histamine receptor H3	7.23	8.70E-08	2.79E-05		
hsp47	heat shock protein 47	7.95	2.34E-06	2.47E-04		
hsp47	heat shock protein 47	12.43	9.08E-07	1.38E-04		
**lamb1**	**laminin, beta 1 (lamb1)**	**6.04**	**3.88E-06**	**3.28E-04**	**4.786**	**1.92E-33**
LOC555472	Novel protein similar to vertebrate cyclin M2 (CNNM2)	6.54	1.29E-06	1.70E-04		
LOC557081	PREDICTED: similar to type VII collagen (LOC557081)	5.39	3.21E-07	6.73E-05		
LOC557081	PREDICTED: similar to type VII collagen (LOC557081)	5.54	4.58E-07	8.52E-05		
LOC557081	PREDICTED: similar to type VII collagen (LOC557081)	6.00	3.49E-07	7.10E-05		
LOC560546	similar to Solute carrier family 2 member 10 (LOC560546)	8.36	4.39E-08	1.93E-05		
LOC562671	PREDICTED: similar to Reticulocalbin (LOC562671)	5.93	1.38E-08	9.76E-06		
LOC562671	PREDICTED: similar to Reticulocalbin (LOC562671)	7.45	5.28E-09	6.58E-06		
LOC562849	PREDICTED: similar to Coronin, actin binding protein, 1C, transcript variant 1	6.91	4.56E-08	1.95E-05		
LOC562849	PREDICTED: similar to Coronin, actin binding protein, 1C, transcript variant 1	7.30	4.81E-09	6.54E-06		
LOC563353	similar to alpha 1 type XXI collagen precursor (LOC563353)	7.83	3.06E-08	1.63E-05		
marcks	myristoylated alanine rich protein kinase C substrate (marcks)	14.82	6.85E-10	3.90E-06	7.463	4.74E-16
mdka	midkine related growth factor (mdka)	10.30	3.70E 09	6.22E 06		
mdka	midkine related growth factor (mdka)	14.77	6.35E 09	6.84E 06		
mmp13	matrix metalloproteinase 13 (mmp13)	12.73	1.58E-09	4.05E-06		
mmp13	matrix metalloproteinase 13 (mmp13)	16.80	1.14E-09	3.90E-06		
olfm2	olfactomedin 2 (olfm2)	9.22	2.12E-07	5.01E-05		
**pcolce2b**	**procollagen C-endopeptidase enhancer 2b (pcolce2b)**	8.23	6.46E-08	2.41E-05	**3.127**	**1.27E-9**
**pdlim3**	**actinin-associated LIM protein (pdlim3)**	**11.13**	**9.06E-09**	**8.07E-06**	**9.312**	**4.28E-7**
**pea3**	**ETS-domain transcription factor pea3 (pea3)**	**4.06**	**2.03E-06**	**2.27E-04**	**4.597**	**8.85E-11**
pnoc	prepronociceptin (pnoc)	4.30	1.26E-07	3.46E-05		
prss35	protease, serine, 35 (prss35)	8.36	3.00E-08	1.63E-05		
**rcn3**	**reticulocalbin 3, EF-hand calcium binding domain (rcn3)**	17.56	9.81E-09	8.56E-06	**4.379**	**8.66E-5**
rrbp1	ribosome binding protein 1 homolog (dog) (rrbp1)	17.53	2.88E-10	3.14E-06	10.201	3.06E-7
sepn1	selenoprotein N, 1 (sepn1)	8.98	7.51E-08	2.56E-05		
sepn1	selenoprotein N, 1 (sepn1)	10.68	4.30E-08	1.93E-05		
shha	sonic hedgehog a (shha)	15.15	1.55E-07	3.92E-05		
**slc1a4**	**solute carrier family 1, member 4**	**12.08**	**6.19E-09**	**6.84E-06**	**2.363**	**7.32E-6**
sp5	Sp5 transcription factor (sp5)	5.41	4.04E-07	7.94E-05		
TC301719	Tenascin-C, complete	43.75	8.51E-09	7.91E-06	5.608	1.35E-7
TC328915	Spalt 1, partial (9%)	5.50	8.55E-07	1.30E-04		
TC350566	Actinin-associated LIM protein, partial (45%)	8.02	1.19E-08	9.32E-06		
TC363066	Keratin 18 (Dorsal aorta proneprin kinesin-1), partial (45%)	10.37	4.19E-09	6.31E-06		
tnc	tenascin C (tnc)	21.46	2.48E-09	4.71E-06	5.61	1.35E-7
tubb5	tubulin, beta 5 (tubb5)	41.42	1.93E-09	4.60E-06		
tubb5	tubulin, beta 5 (tubb5)	41.54	2.53E-09	4.71E-06		
zic3	zic family member 3 heterotaxy 1 (odd-paired homolog, Drosophila) (zic3)	4.33	9.55E-06	5.65E-04		

Genes are in alphabetical order.

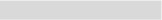
 over expressed in melanoma vs normal.

**Bold** over expressed in melanoma vs other cancers.

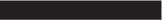
 Gene is associated with poor survival see (figure 6).

### Protein expression in melanoma and normal skin

Protein location, staining intensity and quantity of tumor cells stained (in percent) was inferred from the Protein Atlas database. Protein localization was found to be cytoplasmic or membranous for all, except for BCAN where in addition nuclear staining was observed and for PEA3/ETV4 were exclusive nuclear staining was found (Figure 2A). Staining intensities for proteins were summarized in Figure 2B.

### Oncomine heatmaps: *BAMBI* and *FKBP10*

**Figure 3:**
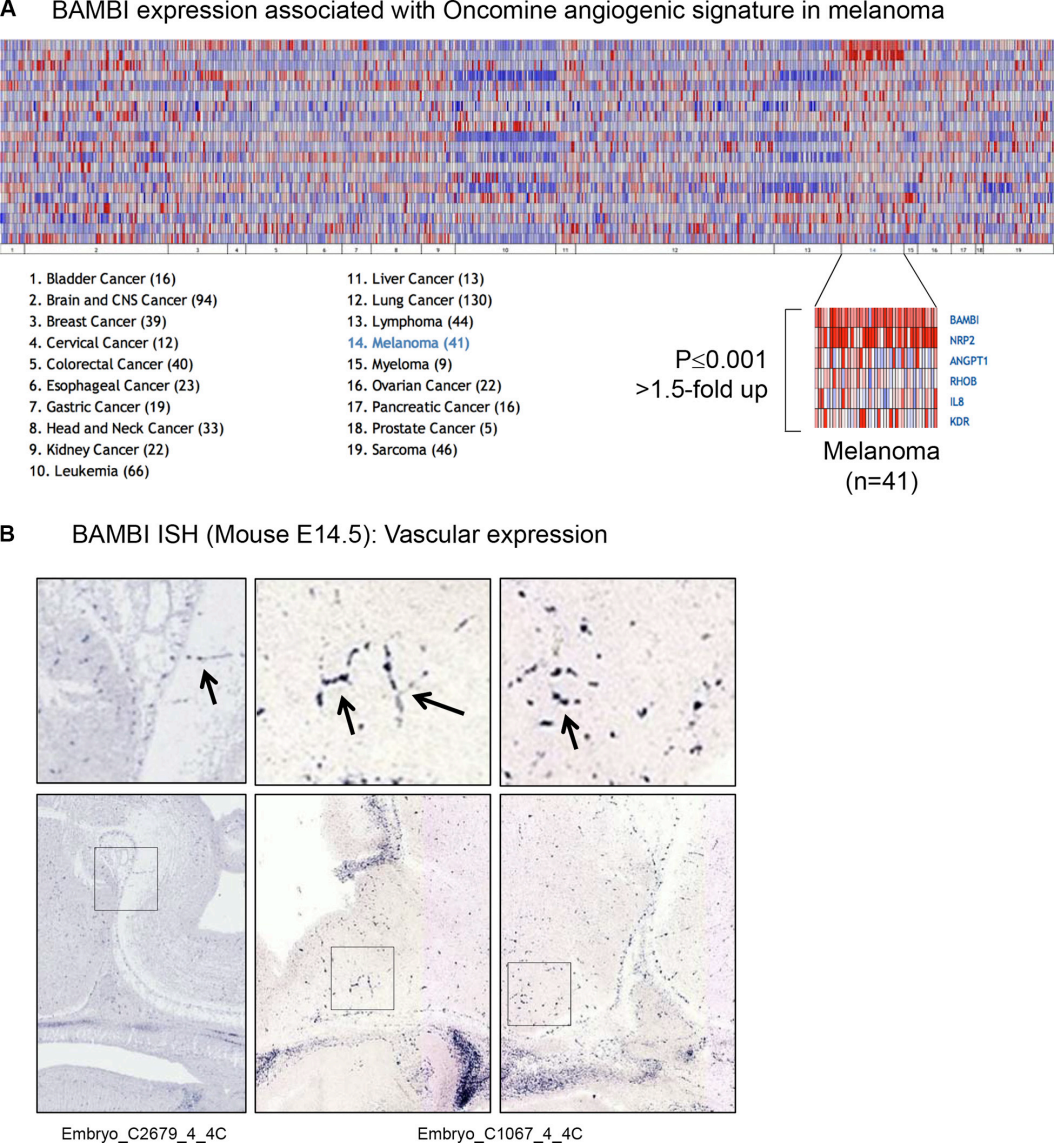
Five genes of the Oncomine angiogenesis gene signature were co-regulated with Bambi transcripts in melanoma compared to other cancers (> 1.5-fold-change, *p* < 0.001) *NRP2*, *ANGPT1*, *RHOB*, IL8 and *KDR*. (**B**) *In situ* hybridization of *BAMBI* mRNA in 14.5 days old mouse embryos shows expression in capillaries of the brain (arrows, magnifications of the square in the lower images).

**Figure 4:**
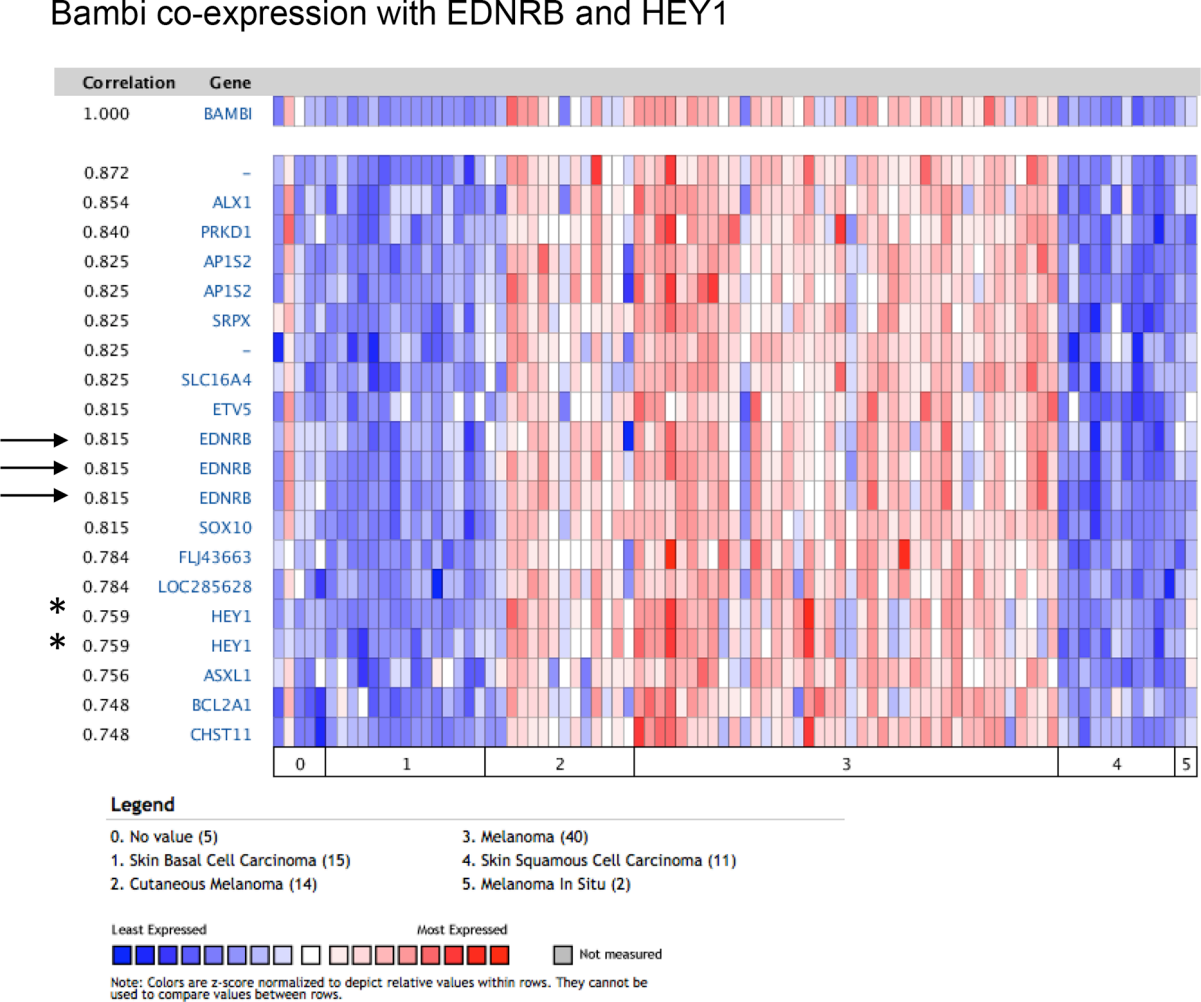
*BAMBI* expression in melanoma strongly correlates with *EDNRB*, encoding for endothelin receptor type B (arrows), an established gene implicated in melanocyte induction and melanoma progression Note also strong correlation with *HEY1* (asterisk), a gene important for embryonic angiogenesis.

*BAMBI* expression (5.556-fold up in melanoma vs normal skin and 10.7-fold vs other cancers) has not been previously associated with melanoma. We compared *BAMBI* expression in a panel of 19 different tumor cell types, and found overexpression most prominent in melanoma cell lines (*n* = 41; Figure 3A). Moreover, when associating a given gene expression pattern with a predefined expression signature of a biological theme such as angiogenesis, five genes with known implication in this process were also upregulated more than 1.5-fold (*P* < 0.001) and co-expressed with *BAMBI*. These genes were *NRP2*, *ANGPTL1*, *RHOB*, *IL8* and *KDR* (Figure 3A). *BAMBI* is also expressed in capillaries in developing mouse embryos, as revealed by *in situ* hybridization (Figure 3B). These data suggest an implication of *BAMBI* expression in melanoma angiogenesis. When compared to other types of skin cancer such as basal cell carcinoma or squamous cell carcinoma, *BAMBI* showed selective overexpression in melanoma and strong co-expression (0.815) with the established melanoma gene *EDNRB*, but also with *HEY1* (0.759), a gene important for vascular development (Figure 4).

**Figure 5:**
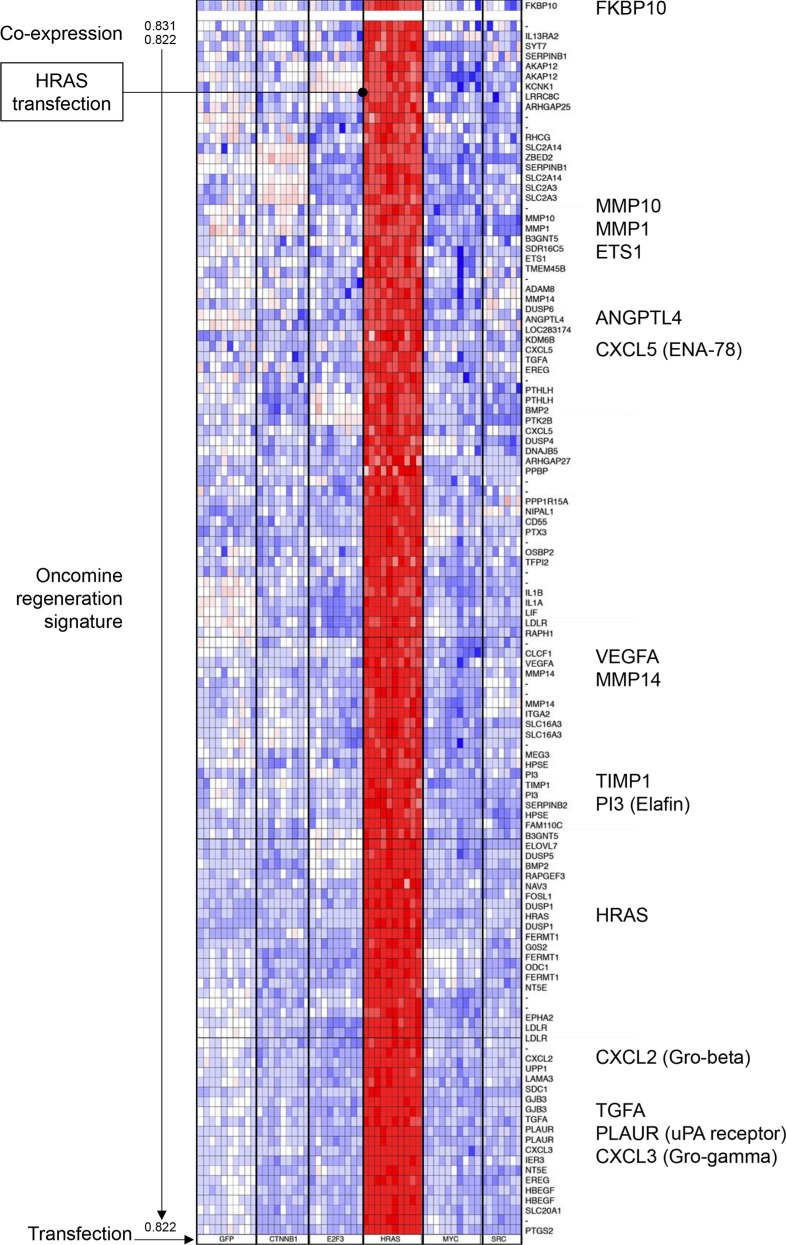
Cluster analysis of the Oncomine regeneration gene signature co-expressed with *FKBP10* gene in transfected human epithelial cells Transfected genes are indicated at the bottom. *FKBP10* is selectively induced in *HRAS*-transfected cells and important known regeneration and angiogenic genes such as *MMP*s, *VEGF* and *CXCL* chemokines are significantly co-expressed with *FKBP10*.

Other regeneration genes overexpressed in melanoma encode for FKBP proteins (FK506-binding proteins). *FKBP7*, *FKBP11* and *FKBP10*, upregulated 3.21, 2.42 and 15.18-fold, respectively, have not yet been described in skin cancer. We focused on *FKBP10* because of its strong expression in melanoma (up 15.18-fold, Table 1). In a co-expression analysis across epithelial cell lines transfected with different oncogenes, *FKBP10* was exclusively associated with *HRAS* transfection (Figure 5). Mice with aberrant *HRAS* activity develop melanoma [10]. Interestingly, when an additional selection criterion was added (Oncomine regeneration gene signature), expression of a number of important genes belonging to chemokine families (*CXCL5*, *CXCL2*, *CXCL3*), proteases (*MMP10*, *MMP1*, *MMP14*) as well as angiogenic genes (*VEGF*, *ANGPTL4*) was very strongly correlated with *FKBP10* expression (0.822). These data suggest that expression of FKBP10 protein might play a role in tissue regeneration, angiogenesis and, as other regeneration genes, in melanoma progression.

### High expression of *FKBP7, DPYSL5 and MDK* is associated with poor patient survival

**Figure 6:**
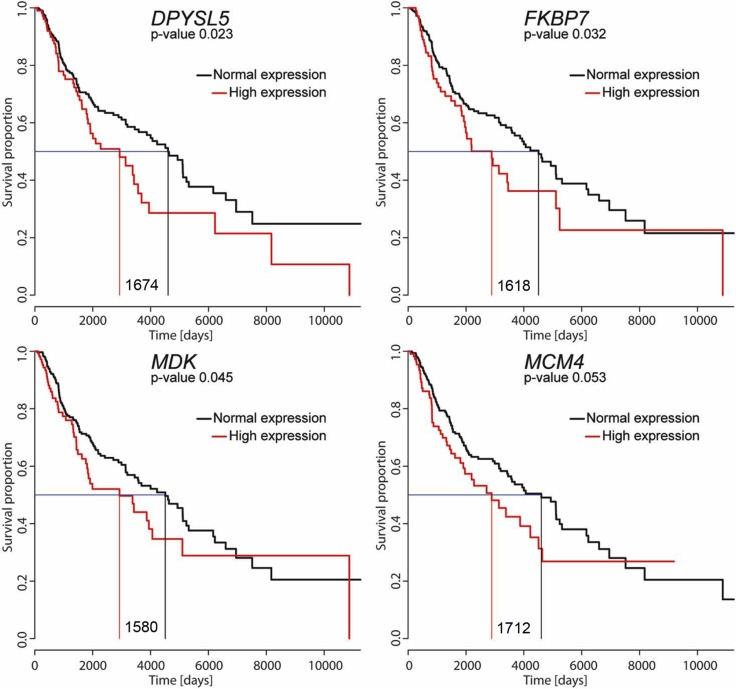
Survival curves of patients with skin cutaneous melanoma For each gene, the red curve represents mortality in patients with a high expression of given gene (expression values above the 75^th^ percentile). The black line represents survival in the remaining patients with lower gene expression levels (0–75^th^ percentile). *P*-values and the median survival for each group are indicated as well as difference in survival (number of days). Note that the *P*-value for the described MM survival gene MCM4 is very close to significance in the TCGA dataset used for this study.

We performed TCGA patient survival analysis integrating all 54 regeneration genes expression. We compared the survival of patients with the highest expression for each gene (75th percentile) with the remaining patients (normal expression). For three genes *DPYSL5*, *FKBP7* and *MDK* we observed significant differences in survival between the two groups (Figure 6). We observed a similar difference in survival for *MCM4* gene, previously reported as a prognostic marker in melanoma [11]. These data suggest that high expression of *DPYSL5*, *FKBP7* and *MDK* can be novel potential prognostic marker for melanoma.

## DISCUSSION

In this paper, we used an *in silico* approach to identify novel genes implicated in cancer. Other computational approaches have recently been developed to model associations of gene networks, metabolic pathways and response to treatment [12]. To evaluate the biological significance of our regeneration data, we compared expression of the most regulated genes to a pathological context. We focused on melanoma, because of strong relationships between melanocyte differentiation during blastema formation and deregulation of melanocyte differentiation program during melanoma progression [2]. Importantly, two genes encoding for known melanoma-promoting proteins were present in our list, such as the extracellular matrix proteins tenascin C (up 5.608-fold) [4] and fibronectin (up 5.654-fold) [13], suggesting that yet unexplored genes participating in blastema regeneration may also have a role in melanoma formation.

The role of BAMBI in melanoma progression has yet to be established. Its expression is inversely correlated with the metastatic potential of melanoma [14]. Oncomine data show that it is expressed by most melanoma cell lines (Figures 3A, 4), but also by capillaries and endothelial cells [15] where it negatively regulates angiogenesis [16]. It might therefore be that BAMBI controls excessive tumor angiogenesis thereby allowing a more efficacious vascularization, similarly to previously described pro- and anti-angiogenic regulators in tumors [17]. Interestingly, Bambi is co-expressed with a well-known melanoma-associated gene, *EDNRB*, important for angiogenesis [2] (Figure 4) suggesting that *BAMBI* is a part of a network of genes regulating vascular growth in melanoma. This idea is further supported by co-expression *BAMBI* with *HEY1*, a downstream effector of Notch signaling important for developmental angiogenesis [18] (Figure 4). Interestingly, Bambi expression in melanoma is regulated by connective tissue growth factor (*CTGF*) since siRNA-mediated knock-down of *CTGF* reduces Bambi expression in melanoma cell lines [19]. Evidence for *BAMBI* implication in melanoma comes mainly from transcript overexpression since protein levels are low (Figure 2B). This may be explained by a rapid degradation of BAMBI protein by lysosomal and autolysosomal mechanisms [15]. *BAMBI* has been found to be part of a gene signature overexpressed in multiple myeloma and pluripotent stem cells [20]. Melanoma stem cells are thought to drive metastasis and tumor progression [21]. It is tempting to speculate that BAMBI participates in melanoma progression via influencing stem cell differentiation or participating in leukocyte mimicry of circulating melanoma cells.

FK506-binding proteins are increasingly discussed as a novel anti-cancer targets [8]. FKBP10 play an important role in human tissue homeostasis because mutations in this gene cause a severe form of osteogenesis imperfecta [22]. Our data suggest that FKBP10 could be a potential target in melanoma treatment, since HRAS specifically induces *FKBP10* expression (Figure 5). *HRAS* overexpression causes melanoma in mice [10] and melanocyte-specific expression of oncogenic *HRAS* has been shown to induce melanoma in zebrafish [23]. HRAS also has an established role in angiogenesis [24].

Other genes whose protein products have evidence of function in cancer include RCN3, RRBP1, PDLIM3 and SLC1A4. Nothing is known about reticulocalbin (RCN3), the protein with highest melanoma expression in the Protein Atlas, but recently, RCN1, a paralog of RCN3, has been found to be a promising tumor marker for renal cell carcinoma [25]. RRBP1 is overexpressed in lung cancer and promotes tumor cell survival [26]. Functions of PDLMI3 in melanoma development can only be speculated; in the adult, the protein is expressed in muscle and plays a role in disorders such as myotonic dystrophy type 1 (DM1) [27] and SLC1A4 has been shown to be induced by the transcription factor MITF in the melanoma cell line SK-MEL-28 [28]. Nuclear expression of PEA3/ETV4 in melanoma may be directly linked to activation of a pro-metastatic program through activation of FAK (Focal adhesion kinase) genes [29].

We identified three genes overexpressed in regenerating blastema that are predictors of poor survival in melanoma patients. As a positive control, we included MCM4, which has been shown to be a poor melanoma survival gene in an independent study [11]. Midkine, a heparin-binding multifunctional growth-promoting protein is overexpressed in various solid tumours and high expression has been shown to be associated with poor survival in breast and lung cancer [30, 31]. The role of midkine in melanoma pathogenesis has not yet been investigated. It is expressed in keratinocytes, not melanoma cells, but recombinant midkine stimulates cell proliferation [32]. It might therefore act as a paracrine growth factor for malignant melanoma cells.

Interestingly, *DPSYL5* (also known as *CRMP5*), a gene which controls brain development, has been shown to promote glioblastoma cell growth and is a potent prognostic factor for this disease, with high expression predicting dismal prognosis [33]. Since melanocytes and glial cells are derived from neural crest precursor cells and share responsiveness to differentiation factors [34], it is possible that the poor prognosis associated with *DPSYL5* in melanoma is due to the common developmental origin with glial cells.

*FKBP7* is upregulated in melanoma compared to other cancers (Table 1). In the paper by Crijns et al., *FKBP7* is described as one of 86 genes associated with low survival in ovarian cancer patients [35]. This is of interest, since proteins of the FKBP family can be targeted by chemical compounds such as rapamycin and its derivatives and constitute therefore promising potential targets for anti-cancer treatment.

Taken together, our data show that genes expressed in a highly controllable system, the zebrafish fin regeneration, recapitulate expression patterns observed in severe human pathologies such as melanoma. Several of the genes and proteins presented here are overexpressed in melanoma and warrant further validation as potential novel melanoma markers or therapeutic targets.

## MATERIALS AND METHODS

### Zebrafish fin amputation and regeneration

Wild-type zebrafish (*Danio rerio*) were purchased from the ZIRC fish center (University of Oregon, USA), and kept in an aquatic holding facility in INSERM zebrafish facilities under standard conditions. Fish housing and handling was performed as described [36]. In brief, fish were stored in tanks for at least 3 weeks before experimentation. The photoperiod was 10-h dark/14-h light cycle and water temperature was maintained at 28 ± 1°C throughout holding and during experiments. Ethical approval for all animal studies was obtained from the institutional animal care and use committee of the INSERM in accordance with the national advisory committee for laboratory animal research guidelines licensed by French authority. For zebrafish fin regeneration assay, adult fish of at least 10 weeks were anesthetized by addition of 0.6 mM tricaine (ethyl-m-aminobenzoate) to water. Caudal fins were amputated at a level proximal to first bifurcation of the bony rays using a scalpel (Figure 1A). After three days, blastema and control fin tissues were harvested and processed for microarrays [37].

### Blastema microarray analysis and statistics

Gene expression of regenerating blastema vs. control fins was analysed by Agilent Zebrafish Oligo Microarray V2 4 × 44K (ref. G2519F) using standard protocols [37]. Overall gene selection procedure is illustrated in Figure 1B. Total RNA was extracted from stump control and regenerated fins using the NucleoSpin^®^ RNA II kit (Macherey-Nagel, Germany), according to the manufacturer's instructions. Purity of RNA samples was evaluated and RNA integrity was controlled on a Bioanalyzer 2100 (Agilent Technologies, USA). Target preparation and hybridization were done according to the manufacturer's instructions. Internal standards were derived from the Two-Color RNA Spike-in Kit (Agilent Technologies, USA). The labeled targets were purified using the RNeasy^®^ Mini Kit (Qiagen) and 825 ng of Cy3- and Cy5-labeled cRNA targets were mixed and incubated on the microarray slides in a rotating oven, at 65°C for 17 hours. Biological samples from three blastema and four normal fin controls (Cy3) were hybridized against a common reference control (Cy5). After washing, slides were scanned using a microarray scanner (Agilent Technologies, USA) at 5-μm resolution and at high and low photomultiplier voltages to optimize the dynamic range of image quantification. The data were extracted from these images using the Agilent Feature Extraction v.9.5.3 software. Data were analyzed with *limma* R/Bioconductor package [38]. Arrays were background corrected using minimum method and a “within-array” loess normalization was performed [39]. Finally, array data were normalized using quantile normalization. We applied the linear modeling approach implemented by *lmFit* and the empirical Bayes statistics implemented by *eBayes* to find genes deregulated in blastema as compared to normal fin. Results were considered statistically significant at *q*-values ≤ 0.05 and fold-change ≥ 4. Microarray data sets have been deposited at NCBI's Gene Expression Omnibus (GEO series accession number GSE58567).

### Protein expression in melanoma and normal skin

The Protein Atlas database (www.proteinatlas.org) [40] was used to retrieve immunhistochemical expression data of melanoma and normal skin.

Below is a table of the patients/antibodies used together with accompanying histological data.

For skin control histology, sections stained by the following antibodies (Protein Atlas abbreviations) were selected: Brevican: HPA007865; Calumenin: HPA006018; Tenascin C: HPA004823; Marcks: CAB022062; FKBP7: HPA008707; FKBP10: HPA051171; FKBP11: HPA041709; S100B: HPA015768; SLC1A4: CAB002757; Reticulocalbin 3: HPA043134; PDZ And LIM Domain Protein 3: HPA004749; RRBP1: HPA009026. Bambi: HPA10866. No useful normal skin data were found for PEA3/ETV4.

### Oncomine gene expression analysis

To focus on genes, which were consistently upregulated in regenerating blastema, we applied a cut-off value of 4. We then used Oncomine™ version 3 and 4.2 (Compendia Bioscience, Ann Arbor, MI, USA [3]) for analysis and visualization of expression data. These data were obtained from the Riker_Melanoma study (*n* = 4 controls; 14 melanoma patients) or the Talantov_Melanoma study (*n* = 7 controls; 45 melanoma patients). The following parameters were included in the search (primary filters): Gene name; Analysis Type: Cancer vs. Normal Analysis and Cancer Type: Melanoma. Expression data of regeneration genes in melanoma compared to othercancers were obtained from the Barrettina_CellLine study (57 melanoma samples vs. 17 other types of cancer), Wagner_CellLine study (18 melanoma samples vs. 3 other cancers), Scherf_CellLine (10 melanoma samples vs. 10 other cancers) and Shankavaram_CellLine 2 study (10 melanoma samples vs. 10 other cancers). The Riker_melanoma study was also used to find genes co-expressed with *BAMBI*.

For concept gene signature studies, we used the following search criteria: Gene: *BAMBI*; Concept: angiogenesis – GO Biological process; Cancer type: melanoma; Concept type: Overexpression. The Garnett_Cellline study was retained for Cluster analysis. For the second concept study we used: Gene name: *FKBP10*; Concept: tissue regeneration – GO Biological process; Analysis type: Co-expression analysis. The Bild_Celline study was retained and group by perturbation was selected (Human Mammary Epithelial Cells Primary Culture). Details on the statistical methods underlying Oncomine have been detailed elsewhere [41].

### *In silico in situ* hybridization

For *in situ* hybridization of Bambi expression, we used the GenePaint database [42], which provides access to high quality *in situ* hybridization images of sections of E14.5 mouse embryos. Capillary expression of BAMBI was demonstrated in the brain.

### Survival analysis of TCGA patients integrating gene expression

Clinical data and RNAseq gene expression for 472 skin cutaneous melanoma samples from the Cancer Genome Atlas (TCGA) were downloaded using FireBrowse (http://gdac.broadinstitute.org/) and normalized with *voom* from the *limma* package. Survival of patients with gene expression above the 75th percentile (classified as high expression) was compared to the remaining patients (normal expression) using the package *survival* in R (http://www.R-project.org/).

**Table:** 

Protein	Antibody ID*	Patient	Diagnosis	Patient id
**FKBP7**	HPA008707	M, age 41	MM**, Metastatic site (M-87206)	2112
**FKBP10**	HPA051171	F, age 91	MM, NOS (M-87203)	3887
**FKBP11**	HPA041709	F, age 52	MM, NOS (M-87203)	4018
**TNC**	HPA004823	M, age 53	MM, NOS (M-87203)	2534
**MARCKS**	CAB022062	F, age 81	MM, NOS (M-87203)	3210
**BAMBI**	HPA10866	M, age 41	MM, Metastatic site (M-87206)	2112
**BCAN**	HPA007865	F, age 30	MM, Metastatic site (M-87206), Pancreas (T-59000)	2598
**PDLIM3**	HPA004749	M, age 59	MM, Metastatic site (M-87206), Lymph node (T-08000)	1414
**RCN3**	HPA043134	M, age 84	MM, NOS (M-87203)	4028
**SLC1A4**	HPA034964	F, age 64	MM, NOS (M-87203)	4026
**CALUA**	HPA006018	F, age 56	MM, Metastatic site (M-87206), Skin (T-01000)	1220
**RRBP1**	HPA009026	F, age 81	MM, NOS (M-87203)	3210
**PEA3/ETV4**	HPA005768	F, age 46	MM, NOS (M-87203)	3223
